# Effectiveness of an App for tobacco cessation in pregnant smokers (TOBBGEST): study protocol

**DOI:** 10.1186/s12884-022-05250-5

**Published:** 2022-12-13

**Authors:** Maria Agràs-Guàrdia, Sara Martínez-Torres, Ester Granado-Font, Meritxell Pallejà-Millán, Felipe Villalobos, Demetria Patricio, Francisca Ruiz, Francesc X. Marin-Gomez, Jordi Duch, Cristina Rey-Reñones, Francisco Martín-Luján

**Affiliations:** 1 Department of Primary Care Camp de Tarragona, Primary Care Center Llibertat (Reus – 3, Institut Català de La Salut, Reus, Spain; 2 Primary Healthcare Research Support Unit Camp de Tarragona, Institut Universitari d’Investigació en Atenció Primària Jordi Gol (IDIAP Jordi Gol), C/Cami de Riudoms, 53-55. Reus-43202, Tarragona, Spain; 3 TICS-AP Research Group, Institut Universitari d’Investigació en Atenció Primària Jordi Gol (IDIAP JGol), Barcelona, Spain; 4 Universitat Oberta de Catalunya (UOC), Barcelona, Spain; 5 Department of Primary Care Camp de Tarragona, Primary Care Center Horts de Miró (Reus – 4), Institut Català de La Salut, Reus, Spain; 6 School of Medicine and Health Sciences, Universitat Rovira I Virgili, Reus, Spain; 7 Fundació Institut Universitari Per a La Recerca a L’Atenció Primària de Salut Jordi Gol I Gurina (IDIAPJGol), Barcelona, Spain; 8 Department of Primary Care Camp de Tarragona, Atenció a La Salut Sexual I Reproductive (ASSIR), Institut Català de La Salut, Reus, Spain; 9 Primary Healthcare Research Support Unit Catalunya Central, Institut Universitari d’Investigació en Atenció Primària Jordi Gol, Sant Fruitós de Bages, Spain; 10 Health Promotion in Rural Areas Research Group, Gerència Territorial de La Catalunya Central, Institut Català de La Salut, Sant Fruitós de Bages, Spain; 11 Department of Computer Engineering and Mathematics, Universitat Rovira I Virgili (URV), Tarragona, Spain

**Keywords:** Smoking Cessation, Pregnancy, Gamification, Telemedicine, Primary Health Care

## Abstract

**Background:**

Tobacco consumption during pregnancy is one of the most modifiable causes of morbidity and mortality for both pregnant smokers and their foetus. Even though pregnant smokers are conscious about the negative effects of tobacco consumption, they also had barriers for smoking cessation and most of them continue smoking, being a major public health problem. The aim of this study is to determine the effectiveness of an application (App) for mobile devices, designed with a gamification strategy, in order to help pregnant smokers to quit smoking during pregnancy and in the long term.

**Methods:**

This study is a multicentre randomized community intervention trial. It will recruit pregnant smokers (200 participants/group), aged more than 18 years, with sporadically or daily smoking habit in the last 30 days and who follow-up their pregnancy in the Sexual and Reproductive Health Care Services of the Camp de Tarragona and Central Catalonia Primary Care Departments. All the participants will have the usual clinical practice intervention for smoking cessation, whereas the intervention group will also have access to the App. The outcome measure will be prolonged abstinence at 12 months after the intervention, as confirmed by expired-carbon monoxide and urinary cotinine tests. Results will be analysed based on intention to treat. Prolonged abstinence rates will be compared, and the determining factors will be evaluated using multivariate statistical analysis.

**Discussion:**

The results of this study will offer evidence about the effectiveness of an intervention using a mobile App in smoking cessation for pregnant smokers, to decrease comorbidity associated with long-term smoking. If this technology is proven effective, it could be readily incorporated into primary care intervention for all pregnant smokers.

**Trial registration:**

Clinicaltrials.gov ID NCT05222958. Trial registered 3 February 2022.

**Supplementary Information:**

The online version contains supplementary material available at 10.1186/s12884-022-05250-5.

## Background

Tobacco consumption during pregnancy is the leading preventable cause of morbidity and mortality for both pregnant and the future child. Smoking in pregnancy has detrimental perinatal effects, including placental abruption, congenital malformations, preterm birth, low birth weight and even miscarriage. Moreover, after gestation, it is associated with cardiac and respiratory problems, hyperactivity, obesity and decreased academic performance in the child [1, 2]. Even though, it has been estimated that 12-22% of pregnant women in industrialized countries smoke during pregnancy [3]. In Spain, 28.3% of women in reproductive age smoke daily and between 30–40% of pregnant women are active smokers at the beginning of pregnancy [4, 5]. Although, around 40% of them quit smoking before or during pregnancy, more than 40 or 85% relapse at 6 or 12 months postpartum, respectively [6].

Pregnancy is considered an ideal opportunity to intervene and control tobacco consumption among smokers and their families [4, 7]. However, the lack of knowledge and education about the harmful effects of smoking during pregnancy is related to higher smoking prevalence, having a further impact in people with either lower socio-economic status or educational level [8, 9]. Different preventive strategies for tobacco consumption are carried out by primary care, such as behavioural support or pharmacotherapy [7]. Although these interventions reduce tobacco consumption in the general population, pregnant smokers do not use to request for them [4, 10, 11]. Actually, potential barriers that make smoking cessation difficult in this population include the social stigma and fear of being judged, as well as the negative attitudes towards the support provided by health care. These facts lead to underestimated rates of smoking prevalence [12, 13]. Thus, the development of alternative strategies on smoking cessation support, specifically adapted to pregnant smokers, is needed.

The generalised use of smartphones and the increasing omnipresence of the internet in these devices, prompted the development of health promotion Apps. Spain is one of the European countries’ leaders in the use of smartphones and health-related topics are among the most searched information. In Spain, it is estimated that around 5 million people use Apps, and their average profile corresponds to young people of reproductive age, between 25 and 40 years old [14]. However, the evaluation of efficacy of health-related Apps is still limited [15]. Recent systematic meta-analysis reported that digital interventions containing behaviour change techniques focused on goals and planning could be effective for smoking cessation during pregnancy [16]. In accordance, interventions that include behaviour change through "problem solving", "information about health consequences", "information about social and environmental consequences", "social support", "negative emotions reduction" and "instructions on how to make behaviour change" are more successful in smoking cessation even after delivery [17]. Recently, many Apps have been developed for pregnant smoking cessation in different countries, including the “SmokeFree Baby” App in the UK that was satisfactory valuated by participants [18].

Previous studies from our research team, determined the effectiveness of an intervention based on gamification and implementation of the Tobbstop App for reducing the prevalence of tobacco use in young population [19]. Beside reducing prevalence of tobacco consumption, 57.7% of habitual users from the Tobbstop App were still abstinent at 12 months [20]. Considering the success of this App, it was implemented in a pilot study of a randomised controlled trial in pregnant smokers [21]. This study revealed a higher percentage of pregnant users from the App that continued their abstinence until delivery in comparison to the control group [21]. Nevertheless, further research is needed in this area with larger samples and longer follow-up periods to replicate the findings of this study and to ensure continued abstinence in the postpartum.

Thus, in the present study, we pretend to adapt the Tobbstop App as an intervention for smoking cessation in pregnant smokers and in the long term. The adaptation of the App will be based on educational content, support for smokers, leisure, games, entertainment, and use of the social network itself, based on the clinical practice guidelines for smoker support of the Catalan Institute of Health and the Department of Health [7, 22].

## Methods

### Study objectives

The primary objective of this research is to evaluate the effectiveness of an intervention against tobacco in pregnant smokers based on the Tobbstop App, which has already been validated for smoking cessation in the general population [19, 20].

The specific objectives which have set out are:

1. To determine the effectiveness of the intervention on smoking cessation during pregnancy.
2. To evaluate the effectiveness of the intervention on prolonged abstinence.
3. To analyse the impact of the intervention to reduce daily consumption in pregnant smokers who continue smoking.
4. To determine the impact of the intervention on pregnancy, birth and neonatal health indicators.

### Study design

This study consists on a randomised community controlled clinical trial to evaluate the effectiveness of the intervention on smoking cessation.

**Fig. 1:**
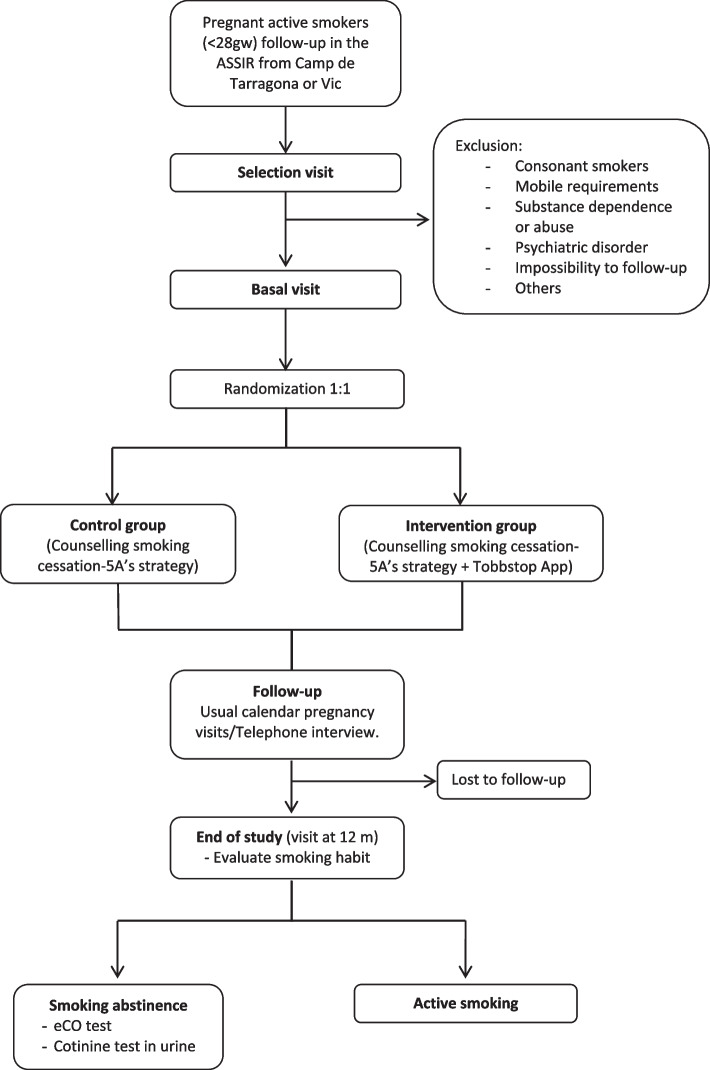
Flowchart of the TOBBGEST study: process of selection, randomization, and follow-up visits

The general framework of this study is shown in Fig. 1 and the activities to be carried out at each visit are detailed in Table 1.

**Table 1:** Diagram of activities to be carried out by participants at each visit

	**Basal visit**	**Usual calendar pregnancy visits**	**Telephone visits**	**Last visit**
**Procedures:**				
Planning day D	X			
Smoking habit	X			
Sociodemographic variables	X			
Clinical history of comorbidities and risk factors associated with tobacco use	X			
Basic physical examination	X	X		
Lifestyles and habits	X	X		
Pregnancy variables	X			
Biochemical measures during pregnancy follow-up		X		
Smoking habit follow-up*		X	X	X
Birth indicators		X		
Newborn health indicators		X		
**Intervention:**				
Control group: 5A’s strategy	X	X	X	X
Intervention group: 5A’s strategy + access to App Tobbstop	X	X	X	X
**Confirmation of smoking abstinence:**				
eCO test		X^*$*^		X^*$*^
Cotinine test in urine				X^*$*^

If the telephone visit and the face-to-face visit coincide, the telephone visit will be omitted

*Relapse. In case of relapse, the following data should be collected: the presence of withdrawal symptoms, if she is still taking the prescribed treatment and if she has smoked a puff

^*$*^eCO and cotinine tests will be taken only to confirm the smoking abstinence reported by the participant

### Study setting

The Sexual and Reproductive Health Care Services (in Catalan, *Atenció a la salut sexual i reproductiva*—ASSIR) is a public Catalan Healthcare service of assistance and educational activities related to orientation and family planning, specific and confidential attention to young people, control and monitoring of pregnancy, among others.

This study will be conducted at the in the ASSIR from the primary care centres of the Camp de Tarragona and Central Catalonia regions (Spain), which are composed of midwives, gynaecologists-obstetricians, nurses, psychologists and administrative personal.

Specifically, pregnancy monitoring centres participating in the recruitment of the study will be four, all of them from the Catalan Institute of Health: three in the Tarragona region (Tarragona, Reus and Valls) and one in the Central Catalonia region (Vic).

All midwives and gynaecologist from the participating centres are invited to collaborate in the study as associate researchers. They will received standardized formation about the methodology of study and the use of App.

### Participant selection

Pregnant smokers visiting the ASSIR centres for any medical reason will be invited to participate if they meet all the inclusion, but none exclusion criteria’s (selection visit).

#### Inclusion criteria

- Pregnant smoker (< 28 gw) aged more than 18 years old.
- Follow-up of pregnancy in the ASSIR units selected.
- Active smokers, who report having smoked sporadically or daily in the last 30 days, at least one cigarette.
- Having a mobile device with Android or iOS operating system and data connection.
- Having skills on the use of Apps and willingness to use them (*).

#### Exclusion criteria

- Consonant smokers or in the Pre-contemplation stage, according to Prochaska and DiClemente’s model [23].
- Having participated in a smoking cessation study within the last 12 months.
- Psychiatric history that prevents or limits follow-up of the study.
- Having another substance dependence or abuse, apart from tobacco consumption.
- Currently under a smoking cessation treatment.
- Impossibility of follow-up participation for any reason.
- Patient refusal to participate or withdrawal from the study.

(*) It is understood that you have skills on the use of Apps and willingness to use them if you answer affirmatively to the following questions:

i. Have you downloaded any application on your mobile?
ii. Would you install an application to help you quit smoking on your mobile?
iii. Have you participated in any chat group?
iv. Have you played any game with your mobile device? (Criteria to ensure the usability of the intervention tool).

### Recruitment, data collection and randomization

#### Recruitment (selection visit)

Participants will be recruited through referrals of midwives from the participating centres. During the selection visit, all pregnant smokers will be advised that smoking during pregnancy could cause detrimental effects for themselves and their foetuses and smoking cessation will be recommended. All eligible participants, will be informed about study characteristics and offered the possibility of participating. If pregnant smoker are interested in taking part of the study, a booklet with additional details will be delivered.

Those eligible participants who agree will be asked to provide signed informed consent and a basal visit will be planned (according to their pregnancy visits) to provide counselling on smoking cessation and the indications to start with the study.

The study is conducted in agreement with the principles of the Helsinki Declaration and the guidelines of Good Clinical Practice. All participants give written informed consent prior to the study enrolment. The protocol has been submitted to the Clinical Research Ethics Committee (CEIC) of the Primary Care Research Institute (IDIAP) Jordi Gol (21/297-P; 15/12/2021) and pending to be approved.

#### Data collection (basal visit)

During the basal visit all the necessary data will be collected using an ad hoc questionnaire (Supplementary data 1) that includes: sociodemographic variables, clinical history of comorbidities and risk factors associated with tobacco use, basic physical examination, lifestyle and habits, smoking habit and characteristics about it, pregnancy variables. Different variables are specifically detailed in Table 2.

**Table 2:** Variables description and registration

**Type of variable**	**Description**	**Description**
**Sociodemographic variables:**		
Age	Date of birth (dd-mm-yyyy)	Basal
Gender	Feminine; Masculine; Non-binary	Basal
National culture	Western; Oriental; Arab; South American; North American	Basal
National culture	A single; In a Heterosexual couple; In a Homosexual couple; Partnered with children	Basal
Social class	British Registrar general's Social Classification	Basal
**Basic physical examination: **		
Weight	Kg	Basal and follow-up
Height	cm	Basal
Blood pressure	mmHg	Basal and follow-up
**Lifestyles and habits: **		
Physical activity	hours/week	Basal and follow-up
Alcohol consumption	units/week	Basal and follow-up
**Clinical history of comorbidities and risk factors associated with tobacco use:**		
Hypertension; Diabetes mellitus; Ischemic heart disease; Cerebrovascular accident; Peripheral arterial disease		Basal
**Smoking habit**		
Nicotine dependence level	Fagerström test: Low ≤ 3; Medium 4-6; High ≥7	Basal
Motivation	Richmond test: Low ≤ 4; Medium 5-6; High ≥ 6	Basal
Stage of change	Prochaska and DiClemente model : Pre-contemplation; Contemplation; Preparation; Action; Maintenance; Relapse	Basal
Consumption before pregnancy	Cigarettes/day	Basal
Current consumption	Cigarettes/day	Basal and follow-up
Type of tobacco consumption	Cigarettes; Cigars; E-Cigarettes; Smokeless Tobacco; Hookah; Pipes; Others	Basal
Age at starting smoking	Years old	Basal
Previous quit attempts	Number	Basal
Reasons for relapse	Weight gain; Stress; Nicotine withdrawal symptoms; Others	Basal
Use of drug therapy or other methods for smoking cessation during the study	Nicotine replacement therapy; Bupropion; Varenicycline; Others	Basal and follow-up
Smokers in the family	Yes; No	Basal
**Pregnancy variables**		
EDD	Estimated Date of Delivery	Basal
TPAL code	Term births; Premature births; Abortions; Living children	Basal
LMP	Last Menstrual Period	Basal
**Biochemical measures during pregnancy follow-up**		
Basic blood tests	Hemogram components (Leukocyte count, erythrocyte count, hematocrit, hemoglobin, mean corpuscular volume, mean corpuscular hemoglobin); Platelets count; Glucose levels	Follow-up (in general 1/trimester)
**Smoking habit follow-up**		
Current consumption	Cigarettes/day	Follow-up
Relapse	Yes; No	Follow-up
Date of Relapse	dd-mm-yyyy	Follow-up
eCO test	<10 ppm, smoking abstinence; >10 ppm, active smoking	Follow-up/End
Cotinine test in urine	<100 ng/ml, smoking abstinence; 100 - 500 ng/ml, occasional smoker; > 500 ng/ml smoker	End
**Birth Indicators **		
Date childbirth	dd-mm-yyyy	Follow-up
Type of childbirth	Eutocic; Dystocic	Follow-up
**Newborn health indicators**		
Body weight	kg	Follow-up
Height	cm	Follow-up
Cranial perimeter	cm	Follow-up
Apgar test	Score: Normal condition 7-10; Moderately depressed 4-6; Severely depressed 0-3	Follow-up
**Data about application usage:**		
Number of days within the app; Number of different days connected; Chat usage		End

The researchers in this study believe that it is appropriate to consider the gender of participants as an interesting factor of study. For this reason, the distinction between gender and sex has been taken into account during the drafting of this study protocol, considering sex as a biological condition and gender as a social perception.

#### Randomization

All participants included in the study will be assigned to the control or intervention group (IG) in a 1:1 ratio using the EPIDAT programme (General Directorate of Innovation and Public Health Management, Xunta de Galicia, Spain; version 3.1). The randomisation will be carried out by the primary care centre and centralised from the Research Support Unit of IDIAP Jordi Gol of Tarragona with a simple numerical randomisation ratio.

### Description of the intervention

All participants will received health education and counselling on smoking cessation, following the 5A’s strategy, which includes five steps: Ask, Advise, Assess, Assist and Arrange. Moreover, each participant, with the support of the referral midwife, will planned a specific day to quit smoking (D-Day).

In addition, participants assigned to the IG will be invited to download the Tobbstop App, either from the Play Store (Android) or Apple's App Store (iOS), along with an explanation of how to use it and the access code will be provided. The IG will have access to the Tobbstop App from 7 days before D-day to 90 days after. Tobbstop App has been successfully tested in the general population [20]. For the present study the contents have been updated and adapted to the study population (pregnant smoker), as described in Supplementary data 2.

### Follow-up and data collection

**Fig. 2:**
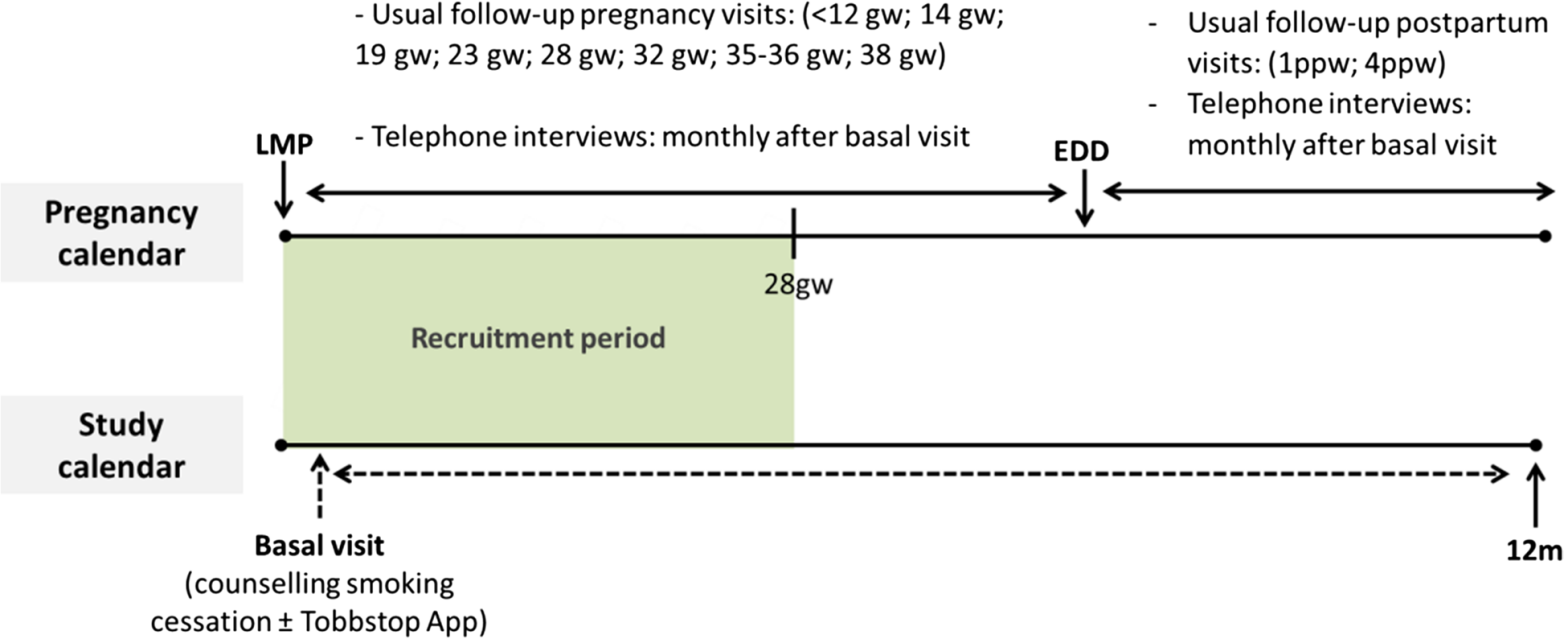
Chronogram of planned follow-up visits during the study and according to the usual follow-up pregnancy visits. LMP, last menstrual period; EDD, estimated date of delivery; gw, gestational week; ppw, postpartum weeks; w,week; m,month. † In case that usual follow-up visits were not possible, telephone interview will be conducted

During the pregnancy period, follow-up of the study will be adapted according to the pregnancy calendar in the ASSIR. If participants did not attend the follow-up visit in the ASSIR, they will be called by phone to ensure participant’s continuance with the study and to evaluate smoking habit (see Fig. 2). If telephone interviews coincide with a pregnancy visit in the ASSIR, the telephone call will be avoided. After the birth, telephone interviews will be planned monthly to evaluate smoking habits in the long term.

Smoking habits will be assessed by questionnaire in the scheduled pregnancy follow-up visits and by telephone interviews. Exhaled carbon monoxide (eCO) will be measured, by an eCO monitor, in every scheduled pregnancy visit.

12 months after the basal visit (when participants receive counsel to quit smoking), smoking habit will be evaluated and those participants reporting smoking abstinence, eCO and urinary cotinine tests will be performed.

Lost to follow-up will be considered [1] when the participant decide to quit the study, [2] when the participant do not attend one of the visits and is impossible to contact with them by telephone call and, [3] when pregnancy follow-up is derived to another healthcare unit (high risk obstetric) or another centre.

### Data recording and storage

The information from the study will be registered and stored in three different platforms:

- Data obtained during the basal and follow-up visits will be registered in a data collection questionnaire (designed specifically for this purpose) and stored in an application accessible from the corporate Intranet of the ICS. Access to this website is restricted and will be controlled by a personal password for each researcher, who will be responsible for entering the records of the participants recruited.
- Data related to clinical history about patient, birth and new-born will be extracted from the Primary Care computerized clinical history (e-CAP).
- Data about App usability, daily smoking progression, emotions, and chat participation, will be extracted from the App and stored in a local service from the URV.

### Outcomes assessment

The main outcome variable will be prolonged abstinence at 12 moths, and the secondary variable will be 3-months and 6-monts prolonged abstinence, and punctual abstinence. The criteria for establishing abstinence have been defined according to the recommendations of the Society for Research on Nicotine and Tobacco [24]:

- Continuous abstinence: sustained abstinence from the time of intervention to a follow-up period.
- Prolonged abstinence: sustained abstinence between an initial grace period (^$^) and a follow-up period.
- Punctual abstinence: abstinence during a window of time (usually 7 days) immediately preceding the time of follow-up.

(^$^) Grace period is the period immediately following the defined quit date or intervention date in which continued smoking is not counted as failure. For most studies it is recommended to be no longer than 2–4 weeks.

To confirm self-reported abstinence, eCO and urine cotinine tests will be taken 12 months after the basal visit. The cut-off point of expired-CO levels is 10 ppm, as lower values indicate non-smoking and higher values indicate smoking in the previous 12–24 h [22]. Urine cotinine values less than 100 ng/mL will reflect an abstinence condition, values between 100 and 500 ng/mL will be accepted as occasional smoker, and values above 500 ng/mL will be considered as smoker [25].

Thus, only those participants reporting smoking abstinence and having eCO levels less than 10 ppm and urine cotinine values less than 100 ng/mL will be considered non-smokers.

### Sample size/power calculation

Accepting an alpha risk of 0.05 and a beta risk of less than 0.2 in a bilateral contrast, 200 subjects will be necessary in each group, in order to detect a statistically significant difference which in the control group is expected to be 0.5 and in the intervention group 0.65. A loss-to-follow-up rate of 5% is estimated. The ARCSINUS approximation (GRANMO Sample Size Calculator v.7.12 available at www.imim.es) was used.

### Blinding

Given the nature of the intervention, it is not possible to mask the participants neither the ASSIR professionals to the study group assignments. The data will be masked for the research team carrying out the statistical analyses.

### Strategy of analysis

#### Preliminary exploration

At the recruitment point of 10% of the total sample, a first data extraction will be carried out to analyze test key elements of the trial, including recruitment and retention strategies, intervention delivery, data collection methods and adherence to the study protocol.

#### Statistical analysis

The efficacy of randomisation will be tested by assessing the comparability and homogeneity of the groups in terms of the similarity of the distribution of the variables of interest at baseline. In addition, the proportion of losses in each group during follow-up will be quantified and assessed as to whether these are independent of the study intervention.

Analysis will be performed as intention to treat. To assess the theoretical bias due to loss to follow-up will be assessed using the 'worst-case analysis' strategy, which will assume that lost patients (in both groups) continue smoking. In case of reporting abstinence but not presenting at the final follow-up visit, self-reported but unconfirmed abstinence will be considered.

Standard statistical tests will be used to analyse the described continuous and categorical variables (e.g. mean, standard deviation, proportions, etc.). Continuous variables will be compared using the Student’s t-test when following a normal distribution or using the U-Man Whitney test if variables are not normally distributed. For categorical variables, Pearson's chi-square test will be used. Point abstinence rates at the end of pregnancy and prolonged abstinence, 12 months after the intervention, will be compared between both groups.

To assess the effectiveness of implementation, odds ratios of association between the allocation group and prolonged abstinence will be calculated. Odds ratios will be calculated crude and adjusted for clinically relevant covariates and relevant interactions between them. Survival analysis will also be carried out for prolonged abstinence at 12 months from counselling smoking cessation. A multivariate Cox regression analysis will be performed comparing the two groups using the Log-Rank test to calculate the Hazard Ratio for abstinence at the end of pregnancy and prolonged abstinence at 12 months after the intervention.

The relationship between missing data and the main variables will be analysed. If missing values exceed 3%, the possibility of applying missing imputation techniques will be studied.

All statistical analysis tests will be conducted with R Statistics package (R foundation for statistical computing, Vienna, Austria; version 4.1.2 or later) and will be considered significant when *p* < 0.05.

#### Patient and public involvement

Patients will be involved in the adaptation of the App. Tobbstop as described in Supplementary data 2.

Results obtained in this study will be disseminated, maintaining privacy and confidentiality of the participants, through publications, reports, and conference presentations. Moreover, at the end of the study, participants will be informed about study results.

## Discussion

The purpose of this study is twofold. Firstly, we aim to test the effectiveness of the Tobbstop App for smoking cessation in pregnant smokers. Secondly, we want to assess the impact of the intervention in pregnancy, birth and neonatal health indicators. Mobile health interventions provide solutions for patient empowerment and facilitate effective behavioural changes in different health areas, including smoking cessation [26].

Tobbstop App has been designed to encourage the general population during the process of smoking withdrawal. Recent results showed its efficacy in smoking cessation in young population [20]. Moreover, a pilot study reported a slightly significant result in increasing smoking abstinence in pregnant smokers [21]. According to these, some Apps with digital smoking cessation interventions designed specifically for pregnant smokers have demonstrated promising results [26]. Particularly, pregnant smokers and women of reproductive age are frequent users of the internet, social media, and mobile Apps, being a prime candidate population for mHealth-supported health care.

Nowadays, there is still a stigma surrounding unhealthy habits during pregnancy, joint to the social belief that pregnancy and motherhood should be a state of happiness. These factors, make more difficult for pregnant smokers searching for help and support to quit smoking, and even they used to lie about their tobacco consumption behaviour [27]. In accordance, offering this intervention through a smartphone App will allow its use without time and place restriction. Thus, ensuring a private and anonymous situation inside the App become an advantage in comparison to other social interventions.

Recent meta-analysis concluded that digital interventions designed specifically for pregnant smokers have a potential effect during pregnancy [28]. However it is still unknown which components are most likely to constitute an effective intervention for smoking cessation. In this study, the adaptation of the Tobbstop App for pregnant smokers includes content based on different topics related to pregnancy and motherhood, achieving a comprehensive intervention with daily activities and practices accompanying pregnant smokers during the process of smoking abstinence. In that sense, focus group interviews have provided in-depth insights into both expertise vision on health promotion and users’ experiences with the Tobbstop App, in order to better understand how smoking cessation smartphone Apps should be designed and configured to fulfil the needs of pregnant smokers.

It is well-known that both pregnancy and postpartum comprise a sensitive period of change and adaptation in which significant physiological changes occur commonly accompanied by the appearance of worries and fears related to the course of pregnancy, childbirth and motherhood [29]. Evidence has shown that the risk to have mental health problems is higher in pregnant smokers in comparison with non-pregnant [30]. In addition, the actual presence of pandemic, sociodemographic and economic crises could lead to change on pregnant behaviours leading to an increase in the prevalence of smoking. Recent data from a qualitative study reported that motherhood frustration as well as partner smoker are key barriers to maintain smoking abstinence during postpartum [31]. Considering these factors, the adaptation of Tobbstop App includes specific sessions of mental health and support, as well as tips to manage feelings and emotions during smoking abstinence, focused on pregnant smokers. Moreover, the fact that volunteers will be followed up for long term, could help to decrease the rate of relapse during postpartum.

High motivation to quit smoking is considered as a key factor to begin and maintain smoke abstinence [32]. A limitation of this study is that participants will be volunteers recruited with high motivation to quit smoking, leading to higher abstinence rates. However, the randomisation of volunteers into intervention and control group will ensure their comparability.

Besides evaluating the effectiveness of the Tobbstop App in pregnant smokers, this study will provide information about sociocultural profile related to higher smoking abstinence as well as data about the App tools with higher adherence and effectiveness. Moreover, if the use of the Tobbstop App proves to be effective, it would be an efficient tool which could be easily extrapolated to other similar contexts.

## Supplementary Information

**Additional file 1.** TOBBGEST study data collection notebook.

**Additional file 2.** Mobile App adaptation.

## Data Availability

The datasets used and analysed during the current study will be available from the corresponding author on reasonable request.

## Abbreviations

APP: Application
ASSIR: Atenció a la Salut Sexual i Reproductiva
eCO: Exhaled Carbon Monoxide
IG: Intervention Group
ICS: Institut Català de la Salut
