# Supplementary material for: Effectiveness of an App for tobacco cessation in pregnant smokers (TOBBGEST): study protocol
Source: BMC Pregnancy Childbirth. 2022 Dec 13;22:933. doi: 10.1186/s12884-022-05250-5 (PMC9745963; doi:10.1186/s12884-022-05250-5)
Supplement: Supplementary file 1 — Additional file 1. TOBBGEST study data collection notebook. [file 12884_2022_5250_MOESM1_ESM.pdf]

TOBBGEST STUDY DATA COLLECTION NOTEBOOK

RECRUITMENT

PATIENT INFORMATION

Birth date: _______________
Date of the last period: _______________

INCLUSION CRITERIA

Pregnant smoker woman: ☐Yes/☐No
The pregnancy will be monitored at the ASSIR: ☐Yes /☐No
Has smoked in the last 30 days: ☐Yes/☐No
Has a smartphone (with Android or iOS operating system): ☐Yes/☐No
Has an internet connection on the phone: ☐Yes/☐No
He regularly uses applications and games on his smartphone: ☐Yes/☐No

EXCLUSION CRITERIA

Suffers from addiction to other psychoactive substances (cocaine, heroin, designer drugs, alcoholism, etc.): ☐Yes/☐No
Suffers from underlying psychotic illnesses (schizophrenia, bipolar disorder, etc.): ☐Yes/☐No
Have you participated in any smoking cessation study in the last 12 months?: ☐Yes/☐No

RICHMOND TEST

Would you like to quit smoking if you could do so easily? ☐Yes/☐No
How interested are you in quitting?

- ☐ None at all
- ☐ A little
- ☐ Pretty much
- ☐ Lots of interest

Will you try to quit smoking in the next few weeks?

- ☐ Definitely not
- ☐ Maybe
- ☐ Yes
- ☐ Definitely Yes

What is the probability that within the next 6 months you will be a non-smoker?

- ☐ None
- ☐ Probably none
- ☐ Probably yes
- ☐ Sure Yes

Name:____________________
Surname: ____________________
Gender:

- ☐ Woman
- ☐ Man
- ☐ Non binary

Mobile Phonel: ____________________
Culture/Nation:

- ☐ Occidental
- ☐ Oriental
- ☐ Arabic
- ☐ South American
- ☐ North American
- ☐ Gypsy
- ☐ Others: ____________________

Civil status:

- ☐ Single
- ☐ Married/ Lives as a couple
- ☐ Widow
- ☐ Divorced
- ☐ Other possibilities

Core coexistence

- ☐ Alone
- ☐ In partnership with a woman
- ☐ In partnership with a man
- ☐ With family
- ☐ Other possibilities

Physical activity (hours/week): ____________________
Alcohol consumption (units/week): ____________________
Level of studies:

- ☐ No education
- ☐ Primary
- ☐ Secondary
- ☐ University studies
- ☐ Master studies
- ☐ Others

Current employment situation (multiple options can be checked):

- ☐ Working
- ☐ Stopped
- ☐ Disability or permanent disability
- ☐ Housewife

☐ Retired
☐ Student
☐ Others. **Specify**: ____________________

Current job, or the last one you had (if you have never had a paid job, specify that of the person on whom you are financially dependent).

☐ Graduates or higher (pharmacists, doctors, lawyers...), company directors and large employers.
☐ Graduates (teachers, nurses, accountants...), small executives, managers and farmers.
☐ Non-manual qualifications (secretary, clerk, city guard, regional police...).
☐ Skilled manual workers (carpenter, plumber, cook...).
☐ Partially qualified (postman, bus driver, machine operator, agricultural workers...).
☐ Unqualified (garbage collector, cleaner, janitor, security guard, day laborers, fishermen, seafood collectors, plain soldier...).
☐ If you are not sure, please specify the job: _______________________________

FIRST VISIT

MODIFIED FAGERSTRÖM TEST

How long does it take after you wake up to smoke your first cigarette?

- ☐ < 5 minutes
- ☐ from 6 to 30 minutes
- ☐ from 31 to 60 minutes
- ☐ > 60 minutes

Do you find it difficult not to smoke in places where it is prohibited?

- ☐ Yes
- ☐ No

Which cigarette is the hardest for you to give up?

- ☐ The first
- ☐ Others

How many cigarettes do you smoke a day?:

- ☐ More than 30
- ☐ From 21 to 30
- ☐ From 11 to 20
- ☐ Less than 11

Do you smoke more in the first hours after getting up than during the rest of the day?:

- ☐ Yes
- ☐ No

Do you smoke, even when he is sick in bed?:

- ☐ Yes
- ☐ No

CONSUMPTION DATA

Date First Visit: _____________

Age of start of consumption (minimum 5 - maximum age of the patient):___________

Number of cigarettes smoked per day (currently and/or in the last 30 days):_____________

Have you tried to quit smoking before?:

- ☐ Yes
- ☐ No

Smokers in the family environment?:

- ☐ Yes
- ☐ No

If you check yes before, degree of kinship, (several options can be chosen):

- ☐ Partner
- ☐ Son/Daughter
- ☐ Father/Mother
- ☐ Sister/Brother
- ☐ Others:___________

Type of tobacco consumed by the participant (multiple options can be chosen):

☐ Traditional cigarettes
☐ Rolling Tobacco
☐ Cigars
☐ Electronic cigarettes
☐ Vaping
☐ Hookah/Shisha
☐ Others. Specify: __________

DAY D PROGRAMMING

What day does he want to stop smoking (the patient must tell us a date): _______________

☐ Written consent has been provided and signed.

Follow-up visit

Visit Day:__________________

Type of visit:

☐ Attended

☐ Telephone

If attended, indicate CO levels (between 0-99)::_____________

Continue with the indicated treatments:

☐ Yes

☐ No

Treatments currently indicated (as of this visit):

☐ Nicotine replacement therapy

☐ Bupropion

☐ Varenicycline

☐ Others (Specify :___________________)

Presence of withdrawal symptoms:

☐ Yes

☐ No

Have you smoked any drag?

☐ Yes

☐ No

Have you had a relapse? (Defined relapse with continued tobacco use for more than 7 consecutive days after a period of abstinence of at least 24 hours or a carboxymetry greater than 10.)

☐ Yes

☐ No

In case of relapse, indicate the reason:

☐ Body weight gain

☐ Stress

☐ Withdrawal symptoms

☐Others. Specify:______________

In case of relapse, indicate the number of cigarettes/day:_____________

Is still pregnant?:

☐ Yes

☐ No

If not, indicate the reason for termination of pregnancy:

☐ Abortion

☐ Eutocic birth (vaginal)

☐ Dystosic Birth (instrumental delivery, caesarean)

In case of delivery, indicate date of delivery:____________

In case of delivery, indicate the baby's first and last name: ______________

In case of delivery, indicate the CIF of the baby:______________
