# Supplementary material for: Effectiveness of an App for tobacco cessation in pregnant smokers (TOBBGEST): study protocol
Source: BMC Pregnancy Childbirth. 2022 Dec 13;22:933. doi: 10.1186/s12884-022-05250-5 (PMC9745963; doi:10.1186/s12884-022-05250-5)
Supplement: Supplementary file 2 — Additional file 2. Mobile App adaptation. [file 12884_2022_5250_MOESM2_ESM.pdf]

MOBILE APP ADAPTATION

Tobbstop App was adapted following a focus group methodology to explore the attitudes, understanding and perceptions of both professional midwives and study population about tobacco cessation. Focus groups allowed communication between research participants in order to better adapt the mobile App to the study population. Two different focus groups were organized:

- **Group of experts:** Focus groups with referrals of midwives and gynecologists from the Sexual and Reproductive Health Care Services (in Catalan, Atenció a la salut sexual i reproductiva - ASSIR).
- **Study group:** Focus groups with people who have smoked during previous pregnancies, to find out their troubles and motivation to quit smoking.

After those meetings, both content and technology characteristics for the App were adapted containing the following information:

- **Information about tobacco effects during pregnancy:** answers to frequently asked questions, advice, suggestions, definitions, illustrations, and images.
- **General information about pregnancy, birth and postpartum**. This area will include:
    - Week to week foetal development: Curiosities about foetal development with image support.
    - Audio-visual material about partum preparation and postpartum. Professional personnel from obstetrics and midwife have developed this content.
- Information about the beneficial **effects of smoking cessation** on the pregnant, foetal and child health.
- Audio-visual support about **mindfulness sessions and respiratory/muscle relaxation exercises.** This material will help pregnant smokers to combat withdrawal symptoms. Professional personnel from obstetrics and midwife areas have developed this audio-visual material.
- **Private social network for participants**: participants can communicate with each other to ask for help, share concerns, or offer help to others.
- **Minigames**: games designed specifically to entertain and educate the participants.
- **Progress registry**: a visual representation of the evolution of the participant's and foetus health progress through the process.
